# Inhaled Methoxyflurane in Australian Emergency Departments—A Cost‐Minimisation Analysis

**DOI:** 10.1111/1742-6723.70326

**Published:** 2026-08-18

**Authors:** Julius Ohrnberger, Ashley Milkop, George Braitberg, Biswadev Mitra, Paul M. Middleton, Sue Anne Yee, Julianne Alexander

**Affiliations:** ^1^ Sapere Research Group Limited Auckland New Zealand; ^2^ Austin Health Heidelberg Victoria Australia; ^3^ Department of Critical Care University of Melbourne Parkville Victoria Australia; ^4^ The Alfred Hospital Melbourne Victoria Australia; ^5^ South Western Emergency Research Institute (SWERI), Liverpool Hospital Liverpool New South Wales Australia; ^6^ South Western Sydney Clinical Campuses, University of New South Wales Warwick Farm New South Wales Australia; ^7^ School of Medicine, Western Sydney University Campbelltown New South Wales Australia; ^8^ Medical Developments International Scoresby Victoria Australia

## Abstract

**Background:**

Around 60% of emergency department (ED) patients present with pain. With growing ED‐demand, there is need for analgesic options that reduce resource use and cost. We estimate the potential cost‐savings from broader use of inhaled methoxyflurane in Australian EDs compared with standard‐of‐care (SOC) analgesics or procedural sedation. The analysis focused on non‐admitted patient presentations requiring analgesia or procedural sedation to treat dislocations, burns, fractures and other injuries.

**Methods:**

We conducted a cost‐minimisation analysis to assess differences in resource use and costs between methoxyflurane, and SOC. Model inputs were informed by a literature review, consultation with ED‐clinicians and Australian Institute of Health and Welfare data, adjusted to 2024/25 population estimates. Costs are reported in 2024/25 Australian dollars, per use case and on aggregate across Australian EDs. Intravenous ketamine, identified by clinicians as the most common comparator, was presented by indication. Sensitivity analyses were undertaken to assess robustness.

**Results:**

Methoxyflurane use could have been associated with lower per‐patient costs than SOC, driven primarily by reduced ED length of stay and staff time. Estimated cost per use case ranged from $62–$283 compared with intranasal fentanyl and $339–$560 compared with intravenous ketamine. At a national level, this equated to potential annual ED‐operational efficiency gains of $9.5–$42.9 million and $34.6–$85.0 million, respectively. Methoxyflurane used for shoulder dislocations contributed an estimated $1.9–$4.8 million when compared to intravenous ketamine.

**Conclusion:**

Broader use of methoxyflurane for the management of pain and minor procedures in Australian EDs could deliver meaningful operational efficiency gains.

## 1 Introduction

Between 60% to 70% of patients present to emergency departments (EDs) with acute pain [1, 2]. Effective and timely pain management is therefore crucial for patient outcomes and treatment [3]. However, the delivery of analgesia is often delayed and sometimes ineffective. Increasing demand on EDs and strain on healthcare resources have intensified the need for analgesic options that are both clinically effective and resource efficient [4, 5, 6, 7].

Methoxyflurane can be used as an inhalational analgesic which is self‐administered by patients for treatment of acute pain associated with trauma or minor surgical procedures, and has been used by Australian emergency services since the early 1970s [7, 8, 9]. Broader adoption of methoxyflurane at Australian EDs may have been be limited by historical concerns regarding nephrotoxicity associated with high‐dose methoxyflurane when used as an anaesthetic [8]. Such adverse effects have not been reported with the substantially lower analgesic doses that are within the scope of this analysis [8, 10]. EDs currently have many analgesic/procedural sedation treatment options and established clinical practices may prevent EDs seeking out potentially more cost‐effective or efficient treatment options. Another potential barrier to wider adoption or inclusion into hospital formularies is the relatively high acquisition cost of methoxyflurane compared with other standard‐of‐care (SOC) analgesic or procedural sedation options. Often, hospitals compare the medicine and associated material cost but have difficulty factoring in staff time or ED bed costs due to the lack of analyses or published research on this issue. Another consideration that may be overlooked is that, compared to nitrous oxide, methoxyflurane is better for the environment, a factor that should be considered, when healthcare facilities consider purchasing arrangements [11].

Methoxyflurane has a rapid onset of action without inducing significant sedation or cardiorespiratory depression and enables a quick recovery [9, 12]. Compared with morphine, fentanyl, ketamine or nitrous oxide used as analgesics or procedural sedation, methoxyflurane requires less intensive and shorter duration clinical monitoring, freeing up clinician time and avoiding the need to use resuscitation or procedure rooms in EDs, which facilitates earlier discharge [7, 9, 13]. Its use has been associated with reductions in ED length of stay (LOS), with one study showing a reduction of 71 min when compared with SOC [14].

These operational characteristics suggest potential for improved patient flow and increased staffing and system efficiencies in ED‐settings in Australia from a broader use of methoxyflurane.

In addition, methoxyflurane is at least non‐inferior, if not more effective when compared to conventional analgesics in terms of moderate to severe pain relief for acute trauma cases, including fractures, burns and dislocations in ED settings [7, 8, 9, 13, 14, 15, 16].

While research supports its clinical utility, the broader cost and resource implications of a potentially increased adoption of methoxyflurane in Australian EDs have not been quantified. Understanding the potential economic impact of broader methoxyflurane use is particularly important in the context of rising ED‐presentations, hospital access block and workforce constraints. Quantifying cost savings could inform decisions about its role in acute pain management and ED‐efficiency.

The objective of this research study was to provide an estimate of the potential healthcare resource utilisation and cost impacts associated with methoxyflurane use compared with SOC in Australian EDs, focusing on non‐admitted patient presentations requiring analgesia or procedural sedation to manage dislocations, burns, fractures and other injuries.

## 2 Method

### 2.1 Study Approach and Perspective

This was an economic evaluation using a cost‐minimisation analysis (CMA) to assess the resource use and cost implications of broader use of methoxyflurane, compared with SOC in Australian EDs.

CMA was undertaken under the assumption of comparable clinical effectiveness between methoxyflurane and SOC, allowing the analysis to focus on differences in resource use and costs rather than clinical outcomes [7, 9, 14, 16].

Model structure, use cases, comparator selection and parameter assumptions were informed by a targeted review of the published literature (Table A1 in Supporting Information) and refined through consultation with emergency medicine clinicians from three tertiary Australian public hospitals in Victoria and New South Wales. The analysis was conducted from the ED‐perspective and thus only includes direct ED‐level costs in 2024/25 Australian dollars. Inpatient costs, downstream health system impacts and patient‐incurred costs were considered out of scope of this analysis.

### 2.2 Identification of Comparators

The types of analgesics and procedural sedation used in Australian EDs vary between hospitals but clinical practice in general reflect the World Health Organisation Analgesic ladder, based on feedback from our panel of clinicians and on published literature [14]. Comparators were selected by a clinical panel to reflect commonly used analgesic and procedural sedation options for the management of moderate‐to‐severe trauma‐related pain in Australian EDs, while also considering the comparators (IV opioids or procedural sedation) used in the Young et al. 2020 study [14]. The comparators chosen were nitrous oxide, IV ketamine, intranasal (IN) fentanyl, IV fentanyl with and without propofol and IV morphine. We note that oral oxycodone and tapentadol, although used for pain management in some settings, were not considered direct comparators for methoxyflurane due to their substantially longer onset of action (up to 30 min) and therefore limited comparability in the acute trauma setting where pain relief is needed quickly or required for a painful procedure [17, 18].

### 2.3 Identification of Volumes and Potential Use Cases

The analysis focused on non‐admitted patients presenting to public Australian EDs with minor trauma requiring analgesia for moderate to severe pain. This population was selected to isolate the impact of analgesic or procedural sedation choice on ED resource utilisation and LOS, independent of inpatient admission costs and to exclude presentations typically requiring longer‐acting analgesia and hospitalisation. Methoxyflurane was evaluated as an alternative analgesic pathway within this population, including presentations where analgesia may facilitate procedures that would otherwise require escalation to procedural sedation.

Based on a literature review and consultations with Australian ED‐clinicians, four clinical indications were identified as relevant use cases for methoxyflurane in Australian EDs: (1) shoulder dislocations [13, 14, 15, 19, 20, 21, 22], (2) fractures [13, 19, 20, 22], (3) acute burns [13, 20, 23] and, (4) injuries such as contusions or lacerations [13, 14, 19, 20, 22]. Ligamentous injuries were considered but excluded following clinician feedback indicating potential for management without analgesia.

National ED‐presentation data for 2023/24 were obtained from the Australian Institute of Health and Welfare (AIHW). Volumes were adjusted to reflect only presentations in patients aged ≥ 5 years, informed by a recent study which found limited use cases and higher admission rates for patients aged < 5 years in Australian EDs [24]. Presentation volumes were then refined to reflect the proportion of cases relevant to the identified indications and the subset of those likely to require analgesia or procedural sedation.

AIHW reports specific patient volumes for non‐admitted burns and other injuries, however, dislocations, ligament and fractures are reported as a single aggregated group. To exclude ligamentous injuries (not a use case for methoxyflurane) from this aggregated group, proportionate adjustment was applied using available NHS England case‐split data [25]. While the case‐mix findings were reviewed and validated by a panel of Australian ED‐clinicians, we acknowledge the extrapolation introduces uncertainty into the modelling.

Further adjustments were made to reflect the proportion of presentations associated with moderate to severe trauma pain. For other fractures and other injuries, this was based on percentage within most complex levels and most costly (Splits A and B in AIHW data) resulting in 11% and 33%, respectively of the total volume being eligible for methoxyflurane treatment. For dislocations, no adjustment was applied, consistent with clinician feedback and published evidence [14]. For burns, 75% of presentations were assumed to require analgesia [26]. Estimated volumes were scaled to 2024/25 using Australian population growth figures and aligned with observed sales of methoxyflurane to Australian hospitals during the same financial year.

### 2.4 Identification of Cost Items

Three cost categories were included in the analysis: (1) product and consumables cost (2), clinician time cost, relating to the time clinicians (nurses and doctors) spend for dispensing, cannulation, administration and monitoring and (3) LOS, relating to the total time the patient spent in ED.

Product and consumable costs were sourced from Medicine Developments International (methoxyflurane) and from a major Australian medical distributor/wholesaler (other analgesics and consumables).

Clinician time cost estimates were informed by input from Australian ED‐clinicians and valued using NSW award wage rates. Cost further include leave and superannuation and for consultants practice cost [27]. Assumptions of time spent by clinician‐type across workflows for each analgesic are presented in Table A2 Supporting Information.

LOS impacts associated with analgesic choice were modelled using data from a recent ED‐study reporting an average 71‐min reduction in LOS for trauma/limb injury patients treated with methoxyflurane versus IV opioids or procedural sedation [14]. For each comparator and indication, the same mean LOS value was applied. This estimate was considered conservative, as the study reported LOS reductions of 180‐min reductions in selected subgroups, such as shoulder dislocations. The reduction of 71 min sits within the lower range of statistically significant LOS estimates of methoxyflurane reported in the literature, ranging from approximately 65–148 min, depending on the SOC comparator to methoxyflurane [21, 28]. We note LOS is also likely influenced by broader organisational and workflow factors within the ED.

LOS reductions were monetised using published ED bed costs ($198.52 per hour, adjusted to 2024/25 Australian dollars [29]), representing the opportunity cost of ED‐capacity. Assumptions and unit costs are presented in Table A2 Supporting Information.

### 2.5 Calculation of Use‐Case and Aggregate Costs

Using the identified unit costs and estimated patient volumes, a CMA model was developed to calculate per‐patient and aggregate annual ED‐costs associated with analgesic use. Methoxyflurane was compared with SOC, including IV/IN fentanyl, IV morphine, IV ketamine, IV fentanyl with propofol and inhaled nitrous oxide. Multimodal analgesic combinations, which are commonly used in ED practice, were not modelled. Although this represents a simplification of routine care, it was considered appropriate for evaluating the relative resource implications and costs associated with the principal analgesic option, allowing differences in resource use to be attributed to individual treatments. Results are presented as incremental cost differences between methoxyflurane and each comparator. For indication‐specific analyses, IV ketamine was selected as the primary comparator, reflecting clinician feedback regarding its widespread use in Australian EDs.

### 2.6 Sensitivity Analysis

Deterministic sensitivity analyses were conducted to reflect uncertainty in key cost drivers including use cases and LOS reduction magnitude. Four scenarios were tested: (1) LOS one‐way sensitivity, downward (−20%): Mean LOS savings and associated cost savings reduced by 20% relative to the base case. (2) LOS one‐way sensitivity, upward (+20%): Mean LOS savings and associated cost savings increased by 20%. (3) LOS extreme sensitivity (−50%): Mean LOS reduction reduced by 50% relative to the base case, reflecting a conservative scenario given LOS is the primary driver of cost differences. (4) Volume sensitivity (−50%): Total case volumes eligible for methoxyflurane substitution were reduced by 50% to reflect uncertainty in real‐world uptake and the extent of substitution across analgesic and procedural sedation pathways.

## 3 Results

### 3.1 Estimated Eligible Use Cases

The total number of non‐admitted ED‐presentations were 6,320,533 in 2023/24, with 2068 reported burns, 388,158 dislocations, ligamentous injuries and fractures and 428,383 other injuries. Table 1 presents the estimated number of eligible methoxyflurane use cases in Australian EDs in 2024/25, which amounts to 151,652. Use cases are driven by fractures with about 65% of use cases, followed by other injuries (25%), shoulder dislocations (5%), other dislocations (4%) and burns (1%).

**TABLE 1:** Estimated potential case volumes of methoxyflurane at Australian EDs in 2024/25.

Type of indication	Total non‐admitted presentations	Percentage likely requiring analgesia or procedural sedation	Total potential presentations
Shoulder dislocations	10,593	100%	10,593
Other dislocations	6935	100%	6935
Fractures	370,630	33%	122,308
Injuries, other	428,383	11%	47,122
Burns	2068	75%	1551
Total	818,609		188,509
Less existing sales of methoxyflurane to Australian hospitals			(36,857)
Net potential use cases			151,652

*Source:* Authors' calculations.

### 3.2 Estimated Cost Per Case

Base case cost estimates per use case are summarised in Table 2. Lowest total costs of $86.85 are observed for methoxyflurane, whereas IV ketamine was associated with the highest estimated cost per use case. Key driver of cost are the impacts on LOS, explaining between 40% and 90% of the total cost of SOC, while cost of clinician time ranges between 10% and 50%. Product and consumable costs represented a smaller proportion of overall costs across all comparators.

**TABLE 2:** Comparison of estimated costs per case for the base case, and split by cost component in 2024/25 Australian dollars.

Standard‐of‐care comparator	Product cost ($)	Cost of clinician time ($)	LOS impact ($)	Total cost ($)
Methoxyflurane	60.00	26.85	n/a	86.85
IV fentanyl	11.57	39.16	Fixed impact per alternative SOC: 234.91	285.64
IV Morphine	10.61	39.16		284.68
IV ketamine	32.80	276.04		543.75
Fentanyl and propofol	22.67	277.52		535.10
Nitrous oxide	14.00	161.13		410.04
IN fentanyl	11.20	20.52		266.63

*Source:* Authors' calculations.

### 3.3 Estimated Per Case Costs and Annual Operational Efficiency Gains

Table 3 presents the results of the CMA comparing methoxyflurane to SOC in Australian EDs in the base case and sensitivity scenarios 1 and 2. Methoxyflurane was associated with lower estimated costs than SOC comparators. The smallest per‐patient cost differences were estimated when compared to IN fentanyl, ranging between $62 and $283, whereas the largest differences were found when compared to IV ketamine, ranging between $339 and $560, reflecting differences in LOS and clinician time requirements.

**TABLE 3:** Potential unit efficiency gains of methoxyflurane relative to comparator analgesic in 2024/25 Australian dollars.

Standard‐ of‐care comparator	Base case	Scenario 1 Reduce LOS benefit by 20%; reduce cost of ED by 20%	Scenario 2 Increase LOS benefit by 20%; increase cost of ED by 20%	Scenario 3 Reduce LOS benefit by 50%
IV fentanyl	198.79	114.22	302.15	81.34
IV morphine	197.83	113.26	301.19	80.38
IV ketamine	456.9	372.33	560.26	339.45
Fentanyl and propofol	448.25	363.68	551.61	330.80
Nitrous oxide	323.19	238.62	426.55	205.74
IN fentanyl	179.78	95.21	283.14	62.33

*Source:* Authors' calculations.

When the estimated per unit cost differences were applied to estimated eligible case volumes for 2024/25, increased use of methoxyflurane in Australian EDs was associated with substantial potential operational efficiency gains per annum, ranging between $9.5 million and $42.9 million for IN fentanyl and up to $34.6 million to $85.0 million for IV ketamine, representing lower and upper ceiling comparators, respectively (see Table 4).

**TABLE 4:** Total potential operational efficiency gains of methoxyflurane relative to comparator analgesic in 2024/25 Australian dollars.

Standard‐of‐care comparator	Base case ($ million)	Scenario 1 ($ million) Reduce LOS benefit by 20%; reduce cost of ED by 20%	Scenario 2 ($ million) Increase LOS benefit by 20%; increase cost of ED by 20%	Scenario 3 ($ million) Reduce LOS benefit by 50%	Scenario 4 ($ million) Reduce inhaled methoxyflurane replacement volume by 50%
IV fentanyl	30.1	17.3	45.8	12.3	15.1
IV morphine	30.0	17.2	45.7	12.2	15.0
IV ketamine	69.3	56.5	85.0	51.5	34.6
Fentanyl and propofol	68.0	55.2	83.7	50.2	34.0
Nitrous oxide	49.0	36.2	64.7	31.2	24.5
IN fentanyl	27.3	14.4	42.9	9.5	13.6

*Source:* Authors' calculations.

Table 5 presents a breakdown of the CMA for IV ketamine by clinical indication. Most annual potential operational efficiency gains result from fractures, ranging between $22.5 million and $55.1 million, followed by other injuries ($8.7 million to $21.2 million), shoulder dislocations ($1.9 million to $4.8 million), other dislocations ($1.3 million to $3.1 million) and burns ($0.3 million to $0.7 million).

**TABLE 5:** Total potential operational efficiency gains: Comparing methoxyflurane with IV ketamine by clinical indication in 2024/25 Australian dollars.

Type of indication	Adjusted Volume	Base Case ($ million)	Scenario 1 ($ million) *Reduce LOS benefit by 20%; reduce cost of ED by 20%*	Scenario 2 ($ million) *Increase LOS benefit by 20%; increase cost of ED by 20%*	Scenario 3 ($ million) *Reduce LOS benefit by 50%*	Scenario 4 ($ million) *Reduce inhaled methoxyflurane replacement volume by 50%*
Shoulder dislocations	8522	3.9	3.2	4.8	2.9	1.9
Other dislocations	5579	2.5	2.1	3.1	1.9	1.3
Fractures	98,395	45.0	36.6	55.1	33.4	22.5
Injuries, other	37,909	17.3	14.1	21.2	12.9	8.7
Burns	1248	0.6	0.5	0.7	0.4	0.3

*Source:* Authors' calculations.

## 4 Discussion

To the authors' knowledge, this is the first study to provide a comprehensive cost and resource utilisation comparison of methoxyflurane versus SOC in hospital‐based ED‐care, incorporating not only product costs but also wider resource inputs, including clinician time and LOS. The analysis estimates potential system‐wide economic value associated with broader adoption of methoxyflurane. Previous studies have primarily focused on clinical effectiveness, with limited consideration of cost and resource utilisation outcomes.

Existing research found that methoxyflurane provides faster pain relief than SOC in moderate‐to‐severe trauma patients in pre‐hospital settings, albeit at higher product cost [30]. Our findings confirm this when only product costs are considered. However, these prior analyses did not account for broader resource implications relevant to EDs such as the economic value of clinician time and shorter length of stays. There may be potential operational benefits from methoxyflurane across broader emergency care settings, including prehospital care but this warrants further investigation.

Nguyen et al. [12] highlighted cost savings when wider treatment costs, including procedural time, were included, reporting AUD 400 savings per colonoscopy in obese patients when treated with methoxyflurane versus sedation with fentanyl and midazolam. However, that study was limited to a hospital procedure and a selected patient group, limiting generalisability to emergency trauma care.

By explicitly incorporating the clinically relevant opportunity cost of time, our analysis provides a more complete assessment of methoxyflurane's economic value, showing that higher product costs can be substantially offset by reductions in clinician time and operational resource use in time‐critical hospital emergency care, resulting in improved resource utilisation at both the ED and hospital system level.

### 4.1 Limitations

Several limitations of this analysis warrant consideration. LOS and clinician time were valued as opportunity costs, based on the assumption that capacity released through shorter LOS and reduced clinician involvement would be reallocated to the care of other ED patients. This assumption is pragmatic and consistent with current operating conditions in Australian EDs, where access block and prolonged waiting times are widely reported [31].

Accordingly, results are presented as potential operational efficiency gains rather than direct cost savings. These estimates represent technical efficiency gains arising from improved utilisation of constrained ED resources, rather than directly averted expenditures. To address uncertainty around these assumptions, sensitivity analyses were conducted using wide ranges for LOS reductions, generating plausible ranges for the estimated operational efficiency gains.

There is uncertainty regarding the specific clinical use cases and severity profiles in which methoxyflurane is utilised across Australian EDs. Published evidence provides limited granularity on the distribution of indications, injury severity and procedural versus non‐procedural use in routine practice. Although structured feedback from emergency clinicians was used to inform model assumptions, this input may not fully capture the heterogeneity of practice across all Australian ED settings. To address this uncertainty, additional sensitivity analysis using a conservative scenario of half the base case volume was undertaken.

The analysis was constrained by the lack of nationally disaggregated Australian data on ED presentations by injury type, acuity and the proportion of patients requiring analgesia. As a result, aggregate AIHW data were supplemented with input derived from literature, expert opinion and international sources, including NHS England emergency care statistics. Although such data are commonly used in health economic modelling, these assumptions introduce uncertainty when extrapolating to the Australian context.

Patient suitability and contraindications represent an additional limitation. Methoxyflurane is not appropriate for all patients or clinical scenarios due to contraindications, age restrictions and injury characteristics. Uncertainty remains in having appropriately excluded all other potentially ineligible presentations. The aggregate nature of available data limited the ability to further refine eligibility criteria within the model. The conservative sensitivity analysis of 50% volume reduction was intended to address this uncertainty.

Methoxyflurane has a short analgesic duration, and some patients may require supplementary analgesia or procedural sedation escalation, which are downstream requirements not fully captured in the model. Where methoxyflurane reduces but does not eliminate further analgesia needs, modelled cost offsets may be modestly overstated. The 50% substitution and 50% LOS reduction sensitivity analyses address these uncertainties, reflecting conservative assumptions around eligible patient volumes and scenarios where LOS benefits are more modest due to procedural escalation. Importantly, our estimates are conservative in several respects. Paediatric presentations were excluded beyond the under‐five age group. A mean LOS reduction was applied across indications, despite evidence of substantially larger LOS reductions for specific presentations using methoxyflurane compared to SOC, such as shoulder dislocations [14].

Future research should focus on generating granular real‐world evidence linking analgesic choice to ED operational outcomes in the Australian context. Access to patient‐level data would enable stratified analyses by age, injury type, acuity, and clinical indication, improving estimates of eligibility, LOS and resource use. At a system level, future analyses should assess whether LOS‐related efficiency gains translate into measurable access improvements, such as freeing up of high acuity ED beds and reduced waiting times, ambulance offload delays, or rates of patients leaving without being seen.

## 5 Conclusion

Increasing the use of methoxyflurane in Australian EDs can generate potential operational efficiency gains, through reductions in LOS and clinician time requirements. Integration of methoxyflurane into care protocols for trauma pain and minor procedures within EDs warrants continued evaluation through effectiveness and health economic surveillance.

## Author Contributions

**Julius Ohrnberger**, **Ashley Milkop**, **Sue Anne Yee**, and **Julianne Alexander:** study concept and design. **Sue Anne Yee**, **Julianne Alexander**, **George Braitberg**, **Paul M Middleton**, and **Biswadev Mitra:** acquisition of data. **Julius Ohrnberger** and **Ashley Milkop:** analysis and interpretation of data. **Julius Ohrnberger**, **Ashley Milkop**, **Sue Anne Yee**, and **Julianne Alexander:** drafting of manuscript. **Julius Ohrnberger**, **Ashley Milkop**, **Sue Anne Yee**, **Julianne Alexander**, **George Braitberg**, **Biswadev Mitra**, and **Paul M Middleton:** critical revision. **Julius Ohrnberger**, **Ashley Milkop**, and **Sue Anne Yee:** statistical expertise. **Sue Anne Yee** and **Julianne Alexander:** acquisition of funding.

## Funding

This work was supported by Medical Developments International.

## Conflicts of Interest

The work was commissioned and funded by Medical Developments International Limited. J.O. reports grant money to Sapere Research Group Limited to conduct this research conceived and sponsored by Medical Developments International Limited. J.O. further reports grant money to Sapere Research Group Limited to conduct research—not related to this submission—conceived and sponsored by Novartis New Zealand Limited. A.M. reports grant money to Sapere Research Group Limited to conduct this research conceived and sponsored by Medical Developments International Limited. G.B. is a Section Editor for EMA. B.M. is a Section Editor for EMA. P.M. reports Grant money for investigator‐initiated research. $20,000 for funding of biostatistical/data science support for HOPPM (Hospital Outcomes of Prehospital Pain Management) study. S.A.Y. is employed by Medical Developments International which manufactures a product related to the subject matter. J.A. is employed by Medical Developments International which manufactures a product related to the subject matter.

## Supporting information


**Data S1:** Supporting Information.

## Data Availability

The data that support the findings of this study are available from the corresponding author upon reasonable request.
