# Supplementary material for: Inhaled Methoxyflurane in Australian Emergency Departments—A Cost‐Minimisation Analysis
Source: Emerg Med Australas. 2026 Aug 18;38(4):e70326. doi: 10.1111/1742-6723.70326 (PMC13484226; doi:10.1111/1742-6723.70326)
Supplement: Supplementary file 1 [file EMM-38-0-s001.docx]

# ONLINE SUPPLEMENTARY MATERIAL

## Findings from our literature review

We conducted a rapid review of the literature focussing on potential cost inputs, comparable analgesics to inhaled methoxyflurane in ED settings, use cases, and clinical impacts of methoxyflurane compared to other analgesics. Table A1 summarises the findings.

Table A1: Literature review of identified studies/evidence and key findings

| Title | Authors | Year | Type | Key finding |
| --- | --- | --- | --- | --- |
| The Efficacy of Inhaled Methoxyflurane Versus Intravenous Sedation for the Reduction of Acute Shoulder Dislocation | Chavalit Iemsaengchairat (1) | 2024 | Randomised controlled trial | Methoxyflurane has better efficacy than IV sedation; procedural and recovery times are shorter with Methoxyflurane (a total of 18.9 minutes in savings). |
| Cost-Effective Analgesia: The role of inhaled Methoxyflurane (Penthrox) in Patient-Controlled Analgesia | Akan S, Anthony Edman J, Suleiman M, Khoshaba J, Chellaram V, and Nguyen D (2) | 2024 | Summary of findings | Reduction in returns to theatre with Methoxyflurane, substantial cost savings for treatment of burns patients needing dressing changes. |
| A review of the safety and efficacy of inhaled methoxyflurane as an analgesic for outpatient procedures | C. Jephcott, J. Grummet, N. Nguyen, and O. Spruyt (3) | 2018 | Literature review | Methoxyflurane is safe and effective when used as a short-term low dose analgesic; effective for colonoscopies, bone marrow and prostate biopsies, burns dressings, minor general surgical procedures, other outpatient procedures that might require sedation, obstetric practice. |
| Methoxyflurane Inhalation as an Analgesic for Minor Gynecological, Ambulatory, or Emergency Procedures | CADTH Health Technology Review (4) | 2024 | Rapid literature review | Evidence for safety and efficacy of Methoxyflurane for minor medical procedures, cancer-related procedures (e.g. colonoscopies and biopsies), portacath insertion and removal, shoulder dislocation and acute elbow dislocation. |
| Literature review on the use of methoxyflurane in the management of pain in cancer-related procedures | Samuel Finkelstein, Etinosa Oliogu, Albert Yee, Lauren Milton, Leon Rivlin, Patrick Henry, Tara Behroozian, Edward Chow, and Joel Finkelstein (5) | 2023 | Literature review | Assessed use for prostate biopsy, colonoscopy, removal of brachytherapy rods, and bone marrow biopsy demonstrate "equivalency or superiority", quicker procedural recovery times. |
| The ‘green whistle’: A novel method of analgesia for transrectal prostate biopsy | Jeremy Grummet, Sean Huang, Alex Konstantatos and  Mark Frydenberg (6) | 2012 | Single-arm study | For 42 men undergoing a TRUS-guided biopsy, they were content with the procedure and identified insignificant adverse effects. |
| Inhaled methoxyflurane (Penthrox) use in the outpatient and ambulatory setting: a systematic review | Bibi Zeyah Fatemah Sairally, Prathiba M De Silva, Paul Smith, and T Justin Clark (7) | 2023 | Systematic literature review | Methoxyflurane used for gastroenterology,  gynaecology, haematology, orthopaedics and urology: no serious adverse events; most patients would use again; at worst no significant differences in pain or comfort level, at best significant reduction in pain when compared with placebo or intravenous sedation. |
| Inhaled Analgesia in Dermatologic Settings: A Comprehensive Overview of Methoxyflurane | Leah Johnston, Susan Poelman, Benjamin Barankin, Geeta Yadav, Jaggi Rao, and Andrei Metelitsa (8) | 2024 | Comprehensive Overview | Demonstrated efficacy shown in randomised controlled trials in patients with acute trauma-related pain; and for patients undergoing painful procedures for other indications. |
| Portable inhaled methoxyflurane is feasible and safe for colonoscopy in subjects with morbid obesity and/ or obstructive sleep apnea | Nam Q. Nguyen, Leanne Toscano, Matthew Lawrence, Vinh-An Phan, Rajvinder Singh, Peter Bampton, Robert J. Fraser, Richard H. Holloway, and Mark N. Schoeman (9) | 2015 | Open label comparative study  One-sided trial | 140 patients with morbid obesity who underwent colonoscopy with either Penthrox inhalation (85) or AADS (55) were prospectively assessed. Penthrox associated with shorter procedural time, lower incidence of respiratory desaturation, and earlier discharge. |
| Role of Inhaled Methoxyflurane in the Management of Acute Trauma Pain | Andrea Fabbri,  Germana Ruggiano,  Sergio Garcia Collado,  Agnes Ricard-Hibon,  Umberto Restelli,  Giovanni Sbrana,  Franco Marinangeli,  Alberto Farina, and Frank Coffey (10) | 2020 | Literature review | Effective for acute, moderate to severe trauma pain in adult patients. |
| Analgesic effectiveness of methoxyflurane inhaler during genicular nerve block in knee osteoarthritis: a randomized controlled trial | Saowanee Sawang, Pretimon Kimpee, Wichai Itthichaikulthol, Nuj Tontisirin, Suwimon Limpoon, Rattaphol Seangrung, Koravee Pasutharnchat, and Steven Paul Cohen (11) | 2024 | Randomised controlled trial | For patients with knee osteoarthritis, when combined with a local anaesthetic, Methoxyflurane provided superior pain control with no observable adverse effects. |
| Service Evaluation of Methoxyflurane Versus Standard Care for Overall Management of Patients with Pain Due to Injury | Louise Young, George P. Bailey, and Jayne A. C. McKinlay (12) | 2020 | Evaluation | Mean time in ED reduced by 71 minutes in those patients administered Methoxyflurane; for patients with shoulder dislocation, the reduction was 183 minutes, no reduction for patients with lower limb, hip or pelvic injuries. |
| Analgesic Efficacy, Practicality and Safety of Inhaled Methoxyflurane Versus Standard Analgesic Treatment for Acute Trauma Pain in the Emergency Setting: A Randomised, Open-Label, Active-Controlled, Multicentre Trial in Italy (MEDITA) |  | 2019 | Randomised controlled trial | Methoxyflurane provided superior pain relief to standard analgesic treatment in patients with moderate-to-severe trauma pain and may offer a simple, fast, effective non-opioid treatment option. |
| Trauma pain management in the emergency department: use of methoxyflurane as a patient-administered analgesic | Craig Wood (13) | 2022 | Service development project | Use of Methoxyflurane allows more rapid clinical examinations and earlier discharge from ED. |

**List of References**

1.	Lemsaengchairat C. The Efficacy of Inhaled Methoxyflurane Versus Intravenous Sedation for the Reduction of Acute Shoulder Dislocation. J Southeast Asian Orthop. 2025;49(1):3–8.

2.	Khan, Sema, Anthony Edman J., Suleiman M., Khoshaba J., Chellaram V., Nguyen D. Cost-Effective Analgesia: The role of inhaled Methoxyflurane (Penthrox®) in Patient-Controlled Analgesia [Internet]. Northern Health; 2024. Available from: https://www.nh.org.au/research/research-at-northern-health/news-and-events/research-week/research-week-posters/

3.	Jephcott C, Grummet J, Nguyen N, Spruyt O. A review of the safety and efficacy of inhaled methoxyflurane as an analgesic for outpatient procedures. Br J Anaesth. 2018 May;120(5):1040–8.

4.	CADTH. Methoxyflurane Inhalation as an Analgesic for Minor Gynecological, Ambulatory, or Emergency Procedures. Can J Health Technol [Internet]. 2024 Sept 6 [cited 2025 June 30];4(9). Available from: https://www.canjhealthtechnol.ca/index.php/cjht/article/view/RC1545

5.	Finkelstein S, Oliogu E, Yee A, Milton L, Rivlin L, Henry P, et al. Literature review on the use of methoxyflurane in the management of pain in cancer-related procedures. Support Care Cancer Off J Multinatl Assoc Support Care Cancer. 2023 Mar 24;31(4):232.

6.	Grummet J, Huang S, Konstantatos A, Frydenberg M. The “green whistle”: a novel method of analgesia for transrectal prostate biopsy. BJU Int. 2012 Dec;110 Suppl 4:85–8.

7.	Sairally BZF, De Silva PM, Smith P, Clark TJ. Inhaled methoxyflurane (Penthrox) use in the outpatient and ambulatory setting: a systematic review. BMJ Open. 2025 Feb 17;15(2):e089031.

8.	Johnston L, Poelman S, Barankin B, Yadav G, Rao J, Metelitsa A. Inhaled Analgesia in Dermatologic Settings: A Comprehensive Overview of Methoxyflurane. Skin Ther Lett. 2024 Sept;29(5):1–9.

9.	Nguyen NQ, Toscano L, Lawrence M, Phan VA, Singh R, Bampton P, et al. Portable inhaled methoxyflurane is feasible and safe for colonoscopy in subjects with morbid obesity and/or obstructive sleep apnea. Endosc Int Open. 2015 Oct;3(5):E487–93.

10.	Fabbri A, Ruggiano G, Garcia Collado S, Ricard-Hibon A, Restelli U, Sbrana G, et al. Role of Inhaled Methoxyflurane in the Management of Acute Trauma Pain. J Pain Res. 2020 June 25;13:1547–55.

11.	Sawang S, Kimpee P, Itthichaikulthol W, Tontisirin N, Limpoon S, Seangrung R, et al. Analgesic effectiveness of methoxyflurane inhaler during genicular nerve block in knee osteoarthritis: a randomized controlled trial. Reg Anesth Pain Med. 2024 Aug 21;rapm-2024-105777.

12.	Young L, Bailey GP, McKinlay JAC. Service Evaluation of Methoxyflurane Versus Standard Care for Overall Management of Patients with Pain Due to Injury. Adv Ther. 2020 May 1;37(5):2520–7.

13.	Wood C. Trauma pain management in the emergency department: use of methoxyflurane as a patient-administered analgesic. Emerg Nurse J RCN Accid Emerg Nurs Assoc. 2022 Aug 30;30(5):25–30.

## Unit cost breakdown by analgesic

We present in table A2 the breakdown of costs of analgesics by costs relating to the drug, material/consumables, and the time of clinicians for dispensing, cannulation, administration and monitoring.

Table A2 Unit cost of analgesics by drug cost, material cost and clinician time cost

| Analgesic | Drug cost | Other material Cost | Clinician time costs |
| --- | --- | --- | --- |
| Methoxyflurane | $60.00  (Includes inhaler, AC chamber and vial) | $0 | $26.85   - Registrar - 10 mins procedural time - Nurse- 10 mins procedural time and 5 mins dispensing time |
| IV fentanyl | $2.06 | $9.51  (Includes cannulation device, saline, and needles) | $39.16   - Registrar – 10 mins cannulation time - 2 Nurses – 10 mins dispensing time and 5 mins administration time each. |
| IN fentanyl | $2.06 | $9.14  (Includes atomisation device) | $20.52   - 2 Nurses – 10 mins dispensing time and 2.5 mins administration time each. |
| IV morphine | $1.10 | $9.51  (Includes cannulation device, saline, and needles) | $39.16   - Registrar – 10 mins cannulation - Nurses – 10 mins dispensing time and 5 mins administration time each |
| IV ketamine | $14.60 | $18.20  (Includes cannulation device, saline, gravity line and needle) | $276.04   - Consultant – 20 mins procedural time including monitoring. - Registrar – 20 mins procedural time including monitoring. - 2 Nurses – 10 mins dispensing time, 25 mins procedural time each and 1 nurse additional 60 mins monitoring. |
| Fentanyl & propofol | $3.51 | $19.16  (Includes cannulation device, saline, gravity line and needle) | $277.52   - Consultant – 25 mins procedural time including monitoring. - Registrar – 25 mins procedural time including monitoring. - 2 Nurses – 10 mins dispensing time, 25 mins procedural time each and 1 nurse additional 20 mins monitoring. |
| Nitrous oxide | $1.05 | $12.95  (Includes mask and tubes) | $161.13   - Consultant – 17 mins procedural time including monitoring. - Registrar – 17 mins procedural time including monitoring. - 2 Nurses – 15 mins procedural time each and 1 nurse additional 15 mins monitoring. |

*Source: Medical Developments International for inhaled methoxyflurane cost and major Australian wholesaler cost for the comparator medicines and consumables. Time inputs to calculate cost of clinician time were provided by ED clinicians. Cost includes dispensing, cannulation, administration and monitoring time. Based on clinical assessments and NSW award information for healthcare professional time costs; IV=intravenous, SC=subcutaneous, IN=Intranasal*
